# Phlorotannins from *Ecklonia cava* Regulate Dual Signaling Pathways, IL-17RA/Act1 and ERK1/2, to Suppress Ovarian Cancer Progression and Tumor-Associated Macrophage Activation

**DOI:** 10.3390/md24010012

**Published:** 2025-12-24

**Authors:** Eun-Hye Kim, Hwi-Ho Lee, Jung-Hye Choi, Ji-Hye Ahn

**Affiliations:** 1 Department of Korean Pharmacy, Woosuk University, Wanju 55338, Republic of Korea; kimune4307@gmail.com (E.-H.K.); hhlee4083@kakao.com (H.-H.L.); 2 Department of Biomedical and Pharmaceutical Sciences, Kyung Hee University, Seoul 02447, Republic of Korea

**Keywords:** *Ecklonia cava*, phlorotannins, IL-17RA/Act1 pathway, ERK1/2, ovarian cancer invasion, tumor-associated macrophages, molecular docking, network pharmacology

## Abstract

Background: Marine-derived secondary metabolites such as phlorotannins from the edible brown alga *Ecklonia cava* exhibit diverse bioactivities. However, their mechanisms in inflammation-associated cancer remain insufficiently understood. Methods: This study explored the anticancer potential of three major phlorotannins (dieckol, 7-phloroeckol, and 8,8′-bieckol) through network pharmacology, molecular docking, molecular dynamics simulations, and in vitro validation in SKOV3 ovarian cancer cells and tumor-associated macrophages (TAMs). Results: Computational analyses revealed stable binding of phlorotannins to IL-17RA, with 7-phloroeckol and 8,8′-bieckol preferentially engaging loop-proximal regions of the receptor, while dieckol interacted with spatially distinct residues. In SKOV3 ovarian cancer cells, phlorotannins suppressed migration and invasion by approximately 40 to 60%, accompanied by reduced MMP expression linked to IL-17RA–Act1 signaling attenuation and by increased TIMP1 expression in association with transient ERK1/2 activation. In TAMs, phlorotannins attenuated pro-tumorigenic cytokine production and polarization marker expression, indicating suppression of tumor-supportive immune activity. Conclusions: Collectively, these findings demonstrate that *E. cava*-derived phlorotannins exert anti-metastatic effects through dual regulation of IL-17RA/Act1 and ERK1/2 signaling pathways, offering mechanistic insight into their therapeutic potential against inflammation-driven malignancies.

## 1. Introduction

*Ecklonia cava* (*E. cava*, Lessoniaceae), an edible brown alga abundantly distributed along the coasts of Korea and Japan, is a prolific source of high-molecular-weight phlorotannins such as dieckol, 7-phloroeckol, and 8,8′-bieckol [1,2,3]. These polyphenolic compounds, exclusively synthesized through the polymerization of phloroglucinol units, are well-known for their exceptional antioxidant capacity and versatile bioactivities, positioning them as promising candidates for drug discovery and therapeutic development [4]. The specific phlorotannins in *E. cava* exhibit diverse pharmacological effects, including antioxidative [5], anti-inflammatory [6], neuroprotective [7], and anticancer activities [1,8]. Importantly, their multiple aromatic hydroxyl groups and flexible molecular scaffolds enable interactions with various protein targets, allowing for the simultaneous regulation of several key cellular signaling pathways [9]. This inherent structural and functional plasticity makes *E. cava*-derived phlorotannins an attractive model for studying the intricate regulation of oncogenic signaling mediated by marine polyphenols [8,10].

Ovarian cancer (OVCA) remains one of the most aggressive gynecologic malignancies, with limited improvement in survival over recent decades [11]. The lethality of ovarian cancer largely arises from peritoneal metastasis, characterized by the dissemination of tumor cells within the abdominal cavity and the formation of secondary lesions on mesothelial surfaces [12]. Emerging evidence emphasizes that the tumor microenvironment (TME), particularly inflammation and immune cell crosstalk, plays a decisive role in this metastatic progression [13]. Among immune regulators, tumor-associated macrophages (TAMs) constitute a major component of the TME, promoting tumor cell invasion, angiogenesis, and immune evasion through the secretion of cytokines such as interleukin-6 (IL-6), interleukin-10 (IL-10), and vascular endothelial growth factor (VEGF) [14,15,16].

A central signaling axis implicated in this process is the interleukin-17 (IL-17) pathway, which orchestrates chronic inflammation and enhances cancer cell invasiveness [17]. The IL-17 receptor A (IL-17RA), in complex with the adaptor protein Act1, mediates downstream activation of the mitogen-activated protein kinase (MAPK) and nuclear factor-kappa B (NF-κB) pathways, leading to increased expression of matrix metalloproteinases (MMPs) that facilitate extracellular matrix degradation and metastasis [18,19,20,21]. Persistent activation of this pathway also promotes macrophage polarization toward the pro-tumorigenic M2 phenotype, further amplifying inflammatory signaling in the TME [22]. In ovarian cancer, lymphoid cells producing IL-17 have been shown to mobilize a distinct subset of small peritoneal macrophages enriched for IL-17RA and protumor mediators, which directly support tumor cell growth [23]. Although pharmacological blockade of IL-17 signaling has shown promise in preclinical models [24], the identification of natural marine compounds capable of modulating IL-17 receptor A (IL-17RA)–related signaling remains scarce.

Interestingly, recent studies have demonstrated that phlorotannins can interfere with receptor–ligand interactions and regulate multiple intracellular pathways, including extracellular signal-regulated kinase 1 and 2 (ERK1/2) and phosphatidylinositol 3-kinase (PI3K)/protein kinase B (AKT) signaling [25,26]. The ERK1/2 cascade represents a crucial pathway not only in cell proliferation and survival but also in maintaining epithelial integrity and controlling metastasis-related gene expression [27]. Context-dependent activation of ERK1/2 can exert tumor-suppressive effects, such as enhancing the expression of tissue inhibitors of metalloproteinases (TIMPs) and reducing MMP-mediated invasion [28]. However, the relationship between phlorotannin-mediated IL-17RA modulation and ERK1/2 activation in the context of ovarian cancer has not been fully elucidated.

In this study, we investigated three structurally distinct phlorotannins (dieckol, 7-phloroeckol, and 8,8′-bieckol) from *E. cava* to clarify their regulatory effects on inflammation-associated ovarian cancer progression. By integrating network pharmacology, molecular docking, and molecular dynamics simulations with in vitro validation in ovarian cancer cells and TAMs, we aimed to delineate the dual modulatory actions of these marine secondary metabolites on the IL-17RA/Act1 and ERK1/2 pathways. Through this combined computational–experimental approach, we provide mechanistic insights into how *E. cava* phlorotannins attenuate cancer cell invasiveness and remodel the immune microenvironment. These findings expand the pharmacological relevance of marine polyphenols and highlight their potential as marine bioactive compounds that regulate critical signaling networks involved in inflammation-driven cancer metastasis.

## 2. Results

### 2.1. Phlorotannins Are Predicted to Be Associated with OVCA Metastasis via IL-17 Signaling Pathway

**Figure 1:**
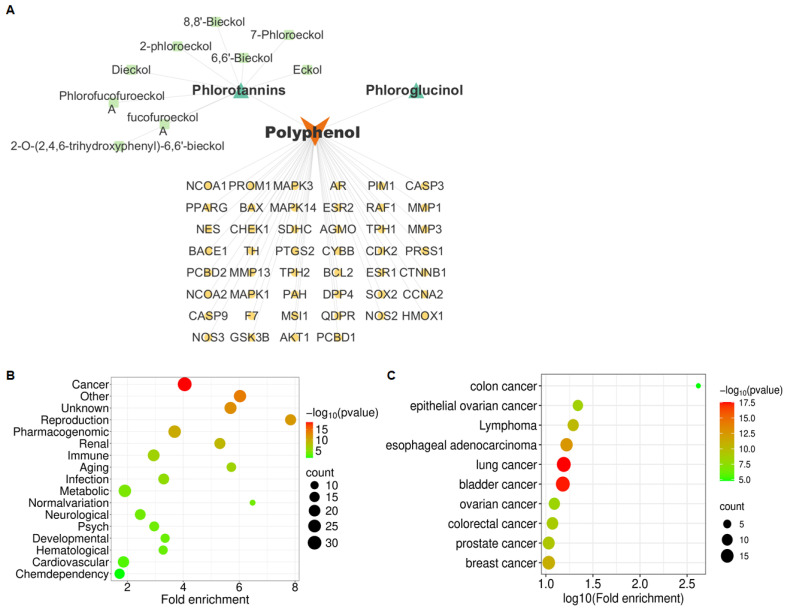
Identification of OVCA as a major phlorotannin-associated disease through GAD analysis: (**A**) Predicted target genes of phlorotannins derived from *E. cava*. (**B**) GAD disease classification showing enrichment of cancer-related categories among predicted targets. GAD disease classification was cut-off of *p* < 0.05. (**C**) Distribution of specific cancer types, with OVCA identified as a major target. GAD disease analysis was performed with targets corresponding to “CANCER” in the GAD disease classification. The top 10 cancer types were selected by setting the threshold *p* < 0.05, and they were sorted in order of count.

Phlorotannins, the predominant phenolic compounds in *E. cava*, are known to exert diverse biological activities, but their role in OVCA remains largely unexplored. To address this, we identified target genes of phlorotannins (Figure 1A) and analyzed them using the genetic association database (GAD) disease analysis. Cancer-related genes represented the largest category among phlorotannin-associated targets (Figure 1B, Table S1). Subsequent GAD analysis restricted to cancer-related targets indicated enrichment of multiple cancer types, including ovarian cancer-related categories such as epithelial ovarian cancer and ovarian cancer, as well as colon-related cancers, as shown in Figure 1C. Although colon cancer exhibited the highest fold enrichment, ovarian cancer was selected as the representative cancer type because a greater cumulative number of associated genes was observed across ovarian cancer-related disease categories. Sixteen OVCA-related genes were identified: NCOA1, PTGS2, PPARG, CTNNB1, ESR1, ESR2, CHEK1, NOS2, AR, MAPK1, MMP3, BCL2, MMP1, CCNA2, DPP4, and CDK2 (Table S2). Additional database screening (HERB for phlorotannin targets; GeneCards for OVCA targets) showed that most overlapping genes matched this set, except for MMP13 and HMOX1. These two genes were included, resulting in 18 genes for further pathway analysis (Table S3).

**Figure 2:**
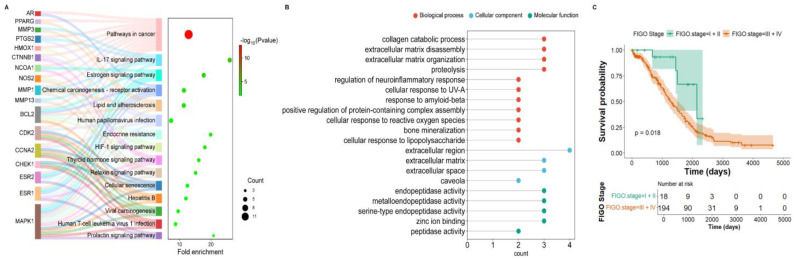
Pathway enrichment and prognostic significance of phlorotannin-responsive genes in OVCA: (**A**) KEGG pathway enrichment analysis of genes shared between phlorotannin targets and OVCA. (**B**) GO enrichment analysis highlighting genes related to the IL-17 signaling pathway. (**C**) Kaplan–Meier survival analysis using TCGA data from 429 patients with available IL17RA expression; high-expression cases (*n* = 212) were stratified by tumor stage (I–II vs. III–IV).

Kyoto Encyclopedia of Genes and Genomes (KEGG) enrichment analysis of the 18 genes showed that “pathways in cancer” contained the largest number of genes, as expected since all were OVCA-related (Figure 2A, Table S4). Notably, the IL-17 signaling pathway displayed the highest fold enrichment (Figure 2A). Gene ontology (GO) analysis of IL-17 pathway-associated genes revealed strong links to metastasis-related processes, including collagen catabolic process, extracellular matrix disassembly, extracellular matrix organization, and proteolysis (Figure 2B, Table S5). In cellular component terms, these genes were enriched in the extracellular region. In molecular function terms, they were related to endopeptidase and metalloendopeptidase activities. Previous studies have reported functional roles for these genes in ovarian cancer progression and IL-17-related signaling pathways, consistent with the present results (Table S6).

Survival analysis using the Cancer Genome Atlas (TCGA) data from 429 patients showed that those with advanced-stage disease (stage III–IV) had higher IL-17RA expression and poorer survival probabilities than early-stage patients (stage I–II) (Figure 2C). Collectively, these findings suggest a possible link between phlorotannins, IL-17 signaling, and OVCA metastasis, which we investigated further in subsequent experiments.

### 2.2. Phlorotannins Attenuate the Migratory and Invasive Ability of OVCA Cells

**Figure 3:**
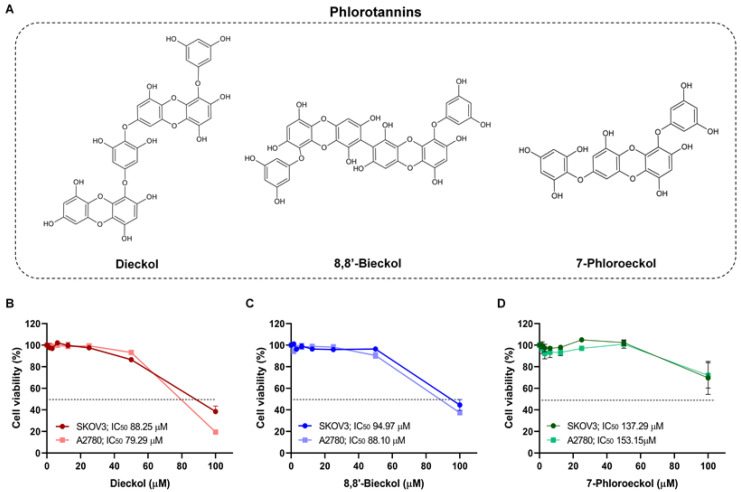
Effects of phlorotannins on OVCA cell viability: (**A**) Chemical structures of dieckol, 8,8′-bieckol, and 7-phloroeckol. (**B**–**D**) MTT assay results showing SKOV3 and A2780 cell viability after 48 h treatment with indicated concentrations of each compound.

**Figure 4:**
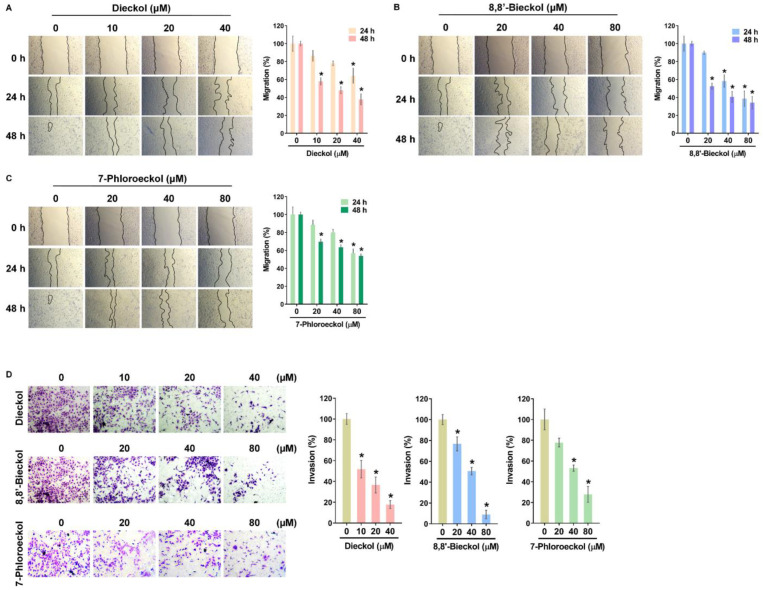
Phlorotannin-mediated suppression of OVCA cell migration and invasion: (**A**–**C**) Wound-healing assay of SKOV3 cells treated with (**A**) dieckol (10, 20, 40 μM), (**B**) 8,8′-bieckol, or (**C**) 7-phloroeckol (20, 40, 80 μM). Migration into the wound area was monitored at 24 and 48 h. (**D**) Transwell invasion assay using Matrigel-coated inserts following 48 h treatment with the same concentrations. Invaded cells were quantified in five random fields per membrane using ImageJ. Representative images from three independent experiments are shown. Data are presented as mean ± SD (*n* = 3). * *p* < 0.05 versus untreated controls.

**Figure 5:**
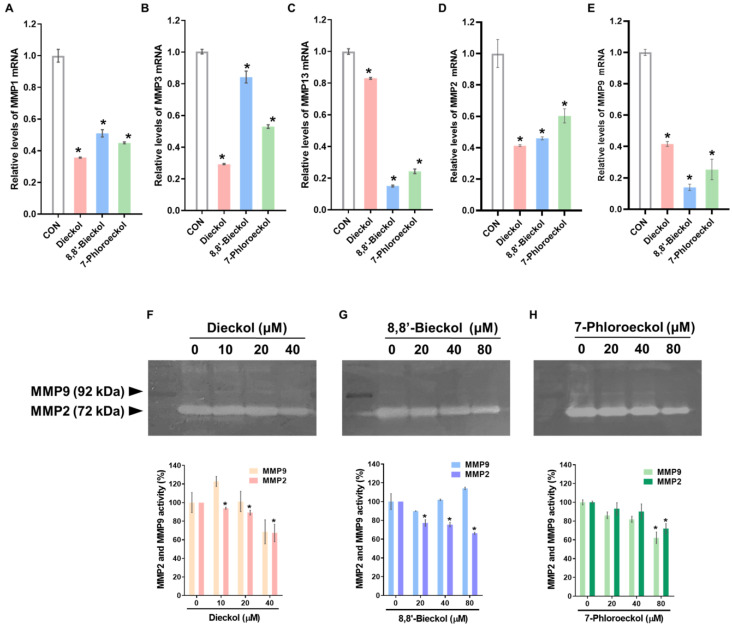
Modulation of MMP expression and activity in SKOV3 cells by phlorotannins: (**A**–**E**) Real-time RT-PCR analysis of MMP1, MMP3, MMP13, MMP2, and MMP9 mRNA expression in SKOV3 cells treated for 48 h with dieckol (40 μM), 8,8′-bieckol (80 μM), and 7-phloroeckol (80 μM). (**F**–**H**) Gelatin zymography analysis of MMP2 and MMP9 enzymatic activity in SKOV3 cells treated for 48 h with dieckol (10, 20, or 40 μM), 8,8′-bieckol (20, 40, or 80 μM), or 7-phloroeckol (20, 40, or 80 μM). Images are representative of three independent experiments. Data are presented as the mean ± SD of three replicates. * *p* < 0.05 versus the untreated group.

We examined the anti-metastatic effects of three phlorotannins (dieckol, 8,8′-bieckol, and 7-phloroeckol) using OVCA cell lines (Figure 3A). Cytotoxicity testing showed that dieckol displayed mild effects, with IC_50_ values of 88.3 ± 4.6 µM in SKOV3 cells and 79.3 ± 0.4 µM in A2780 cells (Figure 3B, Table 1). In contrast, 8,8′-bieckol and 7-phloroeckol exhibited no cytotoxicity at concentrations below 80 µM in either cell line (Figure 3C,D). To assess their impact on metastatic potential, migration and invasion assays were performed using sub-cytotoxic concentrations, including dieckol (10, 20, 40 µM) and 8,8′-bieckol or 7-phloroeckol (20, 40, 80 µM), which were confirmed not to affect cell viability. In wound-healing assays, all three compounds significantly reduced SKOV3 cell migration in a dose-dependent manner (Figure 4A–C). At 24 and 48 h, migratory capacity was reduced by approximately 40 to 60% compared with untreated controls. Similarly, Matrigel-coated transwell assays demonstrated that treatment with dieckol, 8,8′-bieckol, or 7-phloroeckol reduced invasive capacity to approximately 20 to 50% of control levels (Figure 4D). To explore potential molecular mechanisms underlying these effects, we next examined the regulation of MMPs. Based on GO analysis, MMP1, MMP3, and MMP13 were predicted to be associated with OVCA metastasis. Real time RT-PCR confirmed that phlorotannin treatment significantly reduced the mRNA expression of these three MMPs to approximately 30–60% of control levels (Figure 5A–C). Given the established role of MMP2 and MMP9 in OVCA invasion, we further examined their regulation. All three phlorotannins reduced MMP2 and MMP9 mRNA expression to approximately 20–60% of control levels (Figure 5D,E), and gelatin zymography revealed corresponding decreases in enzymatic activity (Figure 5F–H). While dieckol and 8,8′-bieckol did not significantly reduce MMP9 activity, 80 µM 7-phloroeckol markedly suppressed it. All three compounds inhibited MMP2 activity in a dose-dependent manner. Collectively, these findings indicate that phlorotannins can suppress OVCA cell migration and invasion, at least in part, through the downregulation and inhibition of MMPs.

### 2.3. IL-17 Signaling Pathway Is Involved in the Phlorotannin-Inhibited Migration and Invasion of OVCA Cells

**Figure 6:**
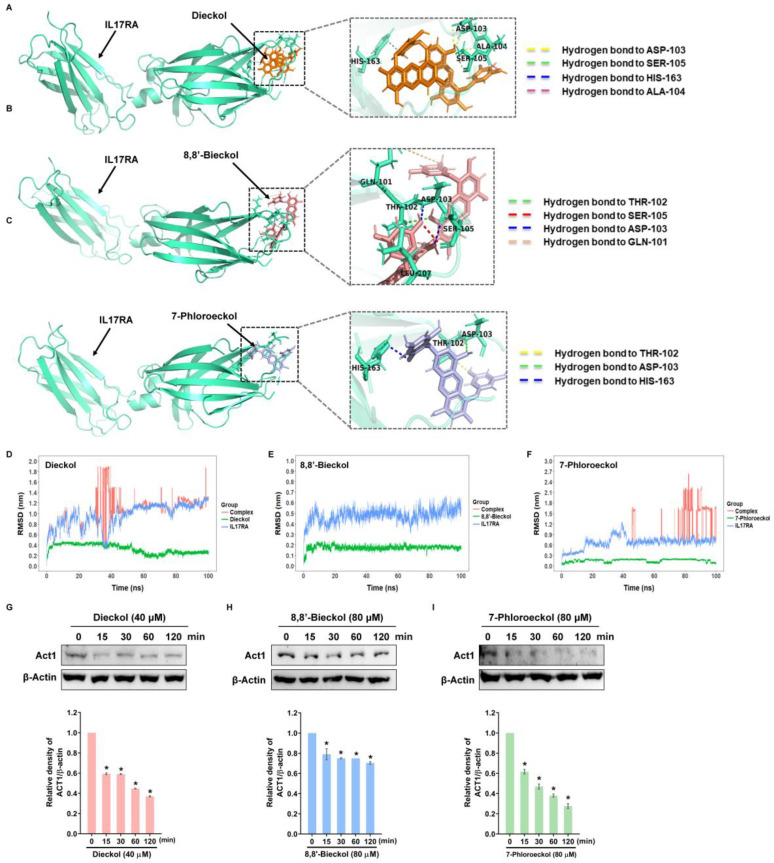
IL-17RA targeting by phlorotannins supported by molecular docking and molecular dynamics: (**A**–**C**) Molecular docking showing binding poses of dieckol, 8,8′-bieckol, and 7-phloroeckol with IL-17RA. (**D**–**F**) RMSD plots from molecular dynamics simulations for each ligand–receptor complex. (**G**–**I**) Western blot analysis of Act1 protein expression in SKOV3 cells treated for 48 h with dieckol (10, 20, or 40 μM), 8,8′-bieckol (20, 40, or 80 μM), or 7-phloroeckol (20, 40, or 80 μM). Data represent the mean ± SD of three replicates. * *p* < 0.05 compared with the untreated group.

KEGG pathway analysis revealed IL-17 signaling as the most enriched pathway in overlapping genes to phlorotannin treatment and OVCA. Based on this, molecular docking simulations were performed to predict the interaction of dieckol, 8,8′-bieckol, and 7-phloroeckol with IL-17RA (Figure 6A–C). All three compounds showed high binding affinity (dieckol: −8.8 kcal/mol, 8,8′-bieckol: −8.5 kcal/mol, 7-phloroeckol: −7.8 kcal/mol; Table 2), indicating strong potential for stable interactions with the receptor. To evaluate binding stability, 100 ns molecular dynamics (MD) simulations were conducted, and root mean square deviation (RMSD) analysis revealed distinct temporal stability profiles for each ligand (Figure 6D–F). The 8,8′-bieckol–IL-17RA complex maintained a consistently low RMSD (~0.5 nm) throughout the simulation, suggesting a stable binding conformation. Dieckol and 7-phloroeckol exhibited greater fluctuations overall but reached transient stable states (dieckol: ~65–70 ns at ~1.1 nm; 7-phloroeckol: ~55–60 ns at ~0.7 nm), indicating temporary conformational stabilization during the simulation. At the most stable simulation frames, 8,8′-bieckol interacted primarily with Asp293 and Trp203, residues positioned away from the main IL-17 anchoring loops (Figure S1). In contrast, dieckol was positioned near Leu107, a residue located within the D1 domain loop that contributes to IL-17A stabilization. 7-Phloroeckol exhibited an intermediate binding profile. The ligand adopted poses near Ser105 and neighboring shallow pockets of the loop boundary region (Figure S1). These binding patterns suggest the potential to interfere with ligand anchoring; however, this interpretation is based solely on computational models and should be experimentally validated. In line with these structural predictions, in vitro experiments showed that all three phlorotannins reduced Act1 expression, a key adaptor molecule in IL-17RA-mediated downstream signaling (Figure 6G–I). These results suggest that phlorotannins may suppress MMP expression and inhibit OVCA cell invasion by blocking IL-17RA-mediated signaling, either through direct disruption of IL-17 binding (7-phloroeckol, 8,8′-bieckol) or through allosteric modulation of receptor conformation (dieckol).

### 2.4. ERK1/2 Pathway Is Involved in Phlorotannin-Inhibited OVCA Cell Invasion

**Figure 7:**
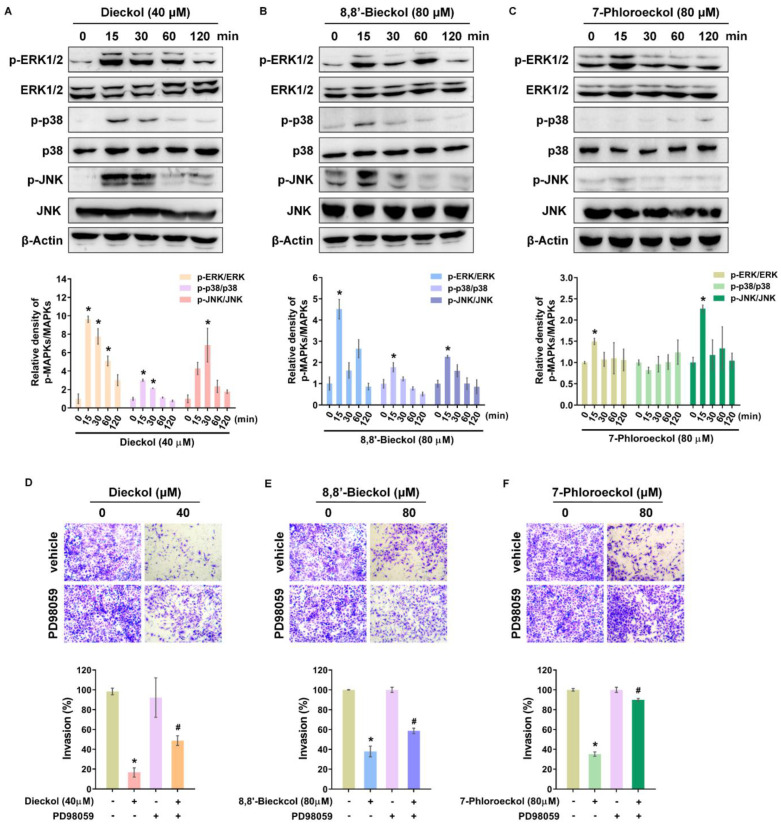
Involvement of ERK1/2 signaling in phlorotannin-inhibited invasion of OVCA cells: (**A**–**C**) Western blot analysis of MAPK activation in SKOV3 cells treated with dieckol (40 μM), 8,8′-bieckol (80 μM), or 7-phloroeckol (80 μM) for the indicated times. (**D**–**F**) Transwell invasion assay following ERK inhibition with PD98059 (10 μM, 4 h) prior to phlorotannin treatment. Quantification was performed in five random fields per membrane using ImageJ. Representative images from three independent experiments. Data are mean ± SD (*n* = 3). * *p* < 0.05 versus untreated; # *p* < 0.05 versus phlorotannin-treated alone.

The MAPK signaling cascade is a well-established downstream effector of IL-17/IL-17R/Act1 signaling, regulating the transcription of various target genes. To assess MAPK activation by phlorotannins, SKOV3 cells were treated with dieckol, 8,8′-bieckol, or 7-phloroeckol, and phosphorylation levels were monitored over 15–120 min. All three compounds rapidly induced phosphorylation of ERK1/2, p38, and c-Jun N-terminal kinase (JNK) at 15 min, followed by a decline toward basal levels at 120 min, consistent with previously reported temporal characteristics of MAPK signaling (Figure 7A–C) [29]. To determine whether MAPK activation contributes to the anti-invasive effects of phlorotannins, invasion assays were performed in the presence of specific MAPK inhibitors (Figure 7D–F and Figure S2). For dieckol and 7-phloroeckol, both ERK1/2 and p38 inhibitors reversed their invasion-suppressive effects. For 8,8′-bieckol, only the ERK1/2 inhibitor restored invasion, while p38 inhibition had no effect. The JNK inhibitor failed to reverse the inhibitory activity of any phlorotannin. Notably, the ERK1/2 inhibitor PD98059 was the only agent that consistently abrogated the anti-invasive effect across all three compounds. These results indicate that ERK1/2 activation serves as a common functional mediator of phlorotannin-induced suppression of OVCA cell invasion, whereas p38 involvement appears compound-specific, and JNK plays no detectable role. Given the observed downregulation of MMP expression and activity by phlorotannins, we next examined whether ERK1/2 activation influences TIMPs. Phlorotannin treatment significantly increased TIMP1 mRNA expression, and this induction was abolished by PD98059 (Figure S3). Collectively, these findings suggest that phlorotannins inhibit OVCA cell invasion through a dual mechanism involving suppression of IL-17/IL-17RA/Act1 signaling to reduce MMP expression and activation of ERK1/2 to enhance TIMP1 expression.

### 2.5. Phlorotannins Attenuate the Pro-Tumoral Activity of TAMs

**Figure 8:**
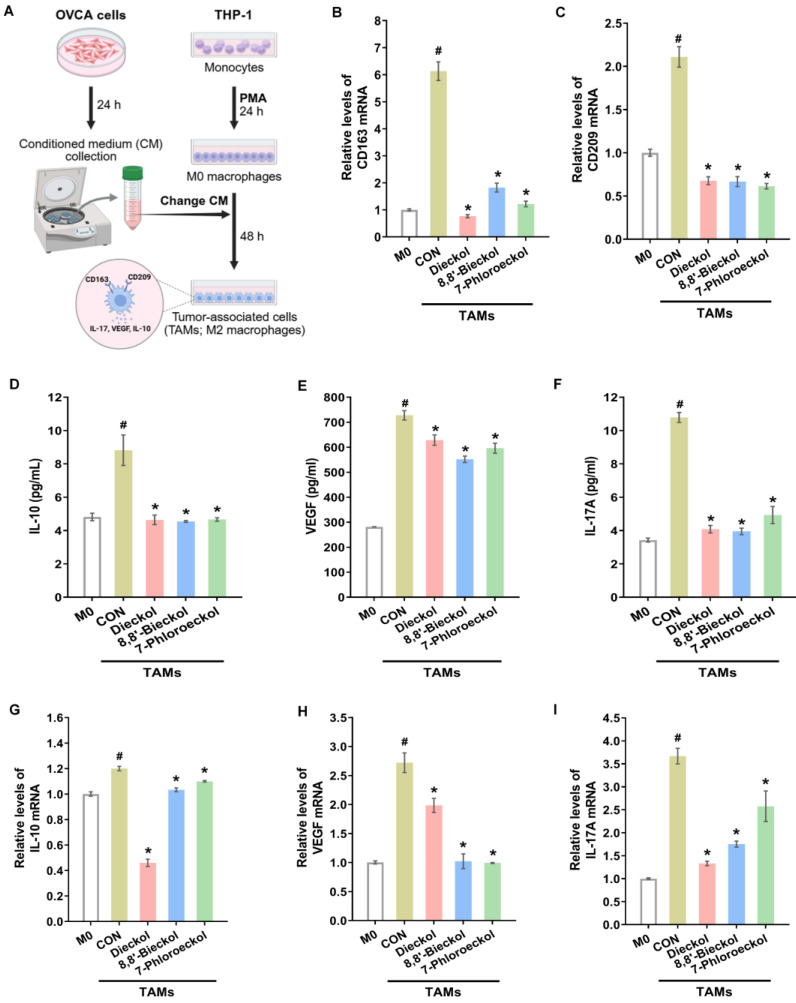
Suppression of pro-tumorigenic TAM activity by phlorotannins: (**A**) THP-1 cells were differentiated into TAM-like cells (M2) using SKOV3-conditioned medium, then treated for 48 h with dieckol (40 μM), 8,8′-bieckol (80 μM), or 7-phloroeckol (80 μM). (**B**,**C**) Real-time RT-PCR analysis of CD163 and CD209 mRNA levels. (**D**–**F**) ELISA measurements of secreted IL-10, VEGF, and IL-17A protein levels. (**G**–**I**) Real-time RT-PCR quantification of corresponding IL-10, VEGF, and IL-17A mRNA expression. Data are mean ± SD (*n* = 3). # *p* < 0.05 versus M0; * *p* < 0.05 versus untreated M2.

Ovarian cancer metastasis within the peritoneal cavity is supported by reciprocal interactions between tumor cells and surrounding stromal and immune cells, particularly TAMs [13]. Tumor cells secrete chemokines such as regulated on activation, normal T cell expressed and secreted (RANTES) and monocyte chemoattractant protein-1 (MCP-1) to recruit macrophages and promote their polarization toward the M2 phenotype, which secretes pro-tumoral mediators including IL-10 and VEGF [30]. To investigate whether phlorotannins modulate TAM recruitment, SKOV3 cells were treated with dieckol (40 μM), 8,8′-bieckol (80 μM), or 7-phloroeckol (80 μM). All treatments significantly reduced RANTES and MCP-1 mRNA levels (Figure S4), suggesting that phlorotannins may limit macrophage infiltration into the tumor microenvironment. Next, we examined whether phlorotannins influence macrophage polarization. M2 polarization was induced using conditioned medium from SKOV3 cells, as confirmed by increased expression of CD163 and CD209 (Figure 8A). Phlorotannin treatment significantly suppressed the induction of both markers in macrophages (Figure 8B,C). Furthermore, phlorotannins reduced both secretion (Figure 8D–F) and mRNA expression (Figure 8G–I) of IL-10, VEGF, and IL-17 from TAMs. Together, these findings demonstrate that phlorotannins interfere with both the recruitment and alternative activation of macrophages, thereby attenuating the pro-tumoral functions of TAMs in the OVCA microenvironment.

## 3. Discussion

The brown seaweed *E. cava* is recognized as a vital source of diverse marine bioactive compounds, prominently featuring phlorotannins, along with sulfated polysaccharides, functional peptides, and carotenoids [31]. As the dominant phenolic constituents, phlorotannins are phloroglucinol oligomers and polymers biosynthesized through the polyketide pathway [32] and have been widely studied for their diverse effects, including anticancer [1,8] and anti-inflammatory activities [6]. Previous studies established that dieckol, a major component, inhibits the proliferation and invasion of MCF-7 breast cancer cells by suppressing MMP9 expression through the inhibition of PI3K/Akt, Wnt/β-catenin, and NF-κB signaling [8]. Furthermore, dieckol suppresses OVCA cell growth by inducing caspase-dependent apoptosis via reactive oxygen species (ROS) generation and regulation of AKT and p38 signaling [1]. Other key components demonstrate comparable biological activities. 8,8′-Bieckol exhibits neuroprotective potential by reducing ROS and apoptosis and inhibiting NO, PGE2, and IL-6 production through the NF-κB pathway suppression [33]. 7-Phloroeckol alleviates alcohol-induced oxidative injury in HepG2 cells by enhancing antioxidant defenses and reducing apoptotic and inflammatory markers [34]. The present work is the first to demonstrate that these three phlorotannins suppress OVCA cell invasion through a dual signaling mechanism involving IL-17RA–Act1 axis inhibition and simultaneous ERK1/2 pathway modulation. The core observation of this study is the paradoxical signaling modulation. Phlorotannins suppressed Act1 expression but concurrently enhanced ERK1/2 phosphorylation. Under canonical physiological conditions, IL-17A binding to IL-17RA recruits the adaptor protein Act1, which then sequentially activates tumor necrosis factor receptor-associated factor 6 (TRAF6) and MAPKs (ERK, p38, and JNK), initiating the transcription of pro-inflammatory and pro-invasive genes [18,35]. Therefore, downregulation of Act1 is typically expected to diminish MAPK activation. The observed increase in ERK1/2 phosphorylation by treatment with phlorotannins, despite Act1 suppression, strongly indicates an alternative, Act1-independent route of activation.

Mechanistically, this complex signaling profile is supported by structural analyses revealing distinct binding behaviors among the three phlorotannins. Docking and molecular dynamics analyses suggest that the three compounds engage IL-17RA through structure-dependent interaction modes rather than a single shared interface. The localization of 8,8′-bieckol within a distal pocket away from the canonical IL-17A anchoring loops raises the possibility of an indirect mode of receptor modulation. By contrast, dieckol and 7-phloroeckol preferentially associate with loop proximal regions of the D1 domain, which have been implicated in stabilizing IL-17A engagement. These spatial differences suggest that phlorotannins may influence IL-17RA signaling through multiple mechanisms, potentially involving alterations in receptor conformation or dynamics rather than direct competition at one binding site. Importantly, these structural findings suggest that phlorotannins interfere with IL-17–IL-17RA coupling through multiple modes of interaction, rather than a singular binding site. These interpretations are currently computational and require confirmation through experimental methods such as site-directed mutagenesis or kinetic binding studies.

This differential binding is critically correlated with the conformational flexibility of the molecules and their resulting differential MAPK network engagement. Dieckol and 7-phloroeckol possess greater conformational mobility, allowing them to transiently interact with diverse scaffold proteins or kinases beyond the IL-17RA–Act1 complex. This plasticity likely facilitates ERK1/2 and p38 activation through alternative upstream regulators, such as Ras-Raf or MEK/MKK modules, which remain functional despite Act1 suppression [36]. This broad engagement explains why the invasion-suppressive effects of both ERK1/2 and p38 inhibitors were reversed for these two compounds. Conversely, 8,8′-bieckol, with its rigid planar configuration, may have a more constrained interaction profile. While all three compounds require ERK1/2 activation for their anti-invasive effect, as demonstrated by the universal abrogation of activity by the ERK1/2 inhibitor PD98059, the structural rigidity of 8,8′-bieckol likely limits its engagement primarily to ERK-associated complexes. This constraint explains why p38 inhibition had no effect on 8,8′-bieckol’s activity. Collectively, the JNK pathway plays no detectable role, but ERK1/2 activation serves as a common functional mediator across all three compounds, whereas p38 involvement appears compound-specific. This highlights how the structural plasticity of phlorotannins underpins their ability to fine-tune intracellular signaling across multiple nodes. Future validation using approaches such as co-immunoprecipitation or in vitro kinase assays will be essential to confirm these proposed modes of pathway engagement.

Another layer of interpretation suggests that these marine compounds may influence upstream membrane events, such as receptor clustering or endosomal trafficking, processes known to govern the amplitude and duration of ERK1/2 signaling [37,38]. Given that phlorotannins possess amphipathic properties and can associate with lipid microdomains, they may stabilize receptor conformations that favor transient rather than sustained ERK1/2 activation. This crucial temporal modulation could further contribute to the observed signaling equilibrium and effectively prevent the pro-survival adaptation that often occurs under therapeutic stress.

Beyond their direct actions on tumor cells, we found that phlorotannins also effectively modulated the tumor microenvironment (TME) by attenuating macrophage recruitment and polarization. The compounds suppressed MCP-1 and RANTES expression in OVCA cells, consequently limiting monocyte infiltration. In TAMs, phlorotannins reduced the expression of M2-like markers (CD163 and CD209) and decreased the secretion of IL-10, IL-17, and VEGF, all of which collectively support tumor progression. By disrupting IL-17-driven crosstalk between TAMs and tumor cells, phlorotannins effectively diminished the inflammatory feedback loop that fosters metastasis. This dual targeting of the tumor and immune compartments underscores their potential as natural agents capable of reprogramming the TME through network-level modulation.

Collectively, this study demonstrates that phlorotannins suppress ovarian cancer cell migration and invasion by up to approximately 60% and attenuate pro-metastatic MMP expression and activity through coordinated modulation of IL-17RA/Act1 signaling and early ERK1/2 activation. To fully transition these mechanistic findings into therapeutic strategies, future work must prioritize functional and translational validation. Subsequent studies should identify and confirm the alternative upstream regulators responsible for the compensatory ERK1/2 activation observed during Act1 suppression. Crucially, the anti-invasive and TME-modulating effects must be verified in immune-competent in vivo models to establish pharmacokinetics, dose–response relationships, and the overall suppression of metastatic spread, thereby guiding the clinical development of these potent marine polyphenols.

## 4. Materials and Methods

### 4.1. Materials

Phlorotannins (dieckol, 8,8′-bieckol, and 7-phloroeckol; >99% purity) were isolated from *E. cava* and kindly provided by Livechem, Inc. (Daejeon, Republic of Korea). The isolation procedures have been described previously [39]. Human OVCA cell lines (SKOV3 and A2780) and the human monocytic cell line THP-1 were obtained from the ATCC (Manassas, VA, USA). RPMI-1640 medium, penicillin, and streptomycin were purchased from Welgene (Gyeongsan, Republic of Korea), and fetal bovine serum (FBS) was obtained from Thermo Fisher Scientific (Waltham, MA, USA). Phorbol 12-myristate 13-acetate (PMA), 2-mercaptoethanol, crystal violet, gelatin, Triton X-100, protease inhibitor cocktail, phosphatase inhibitor cocktail, and Tween 20 were from Sigma-Aldrich (St. Louis, MO, USA). Phenylmethylsulfonyl fluoride (PMSF) was purchased from Roche (Mannheim, Germany). MTT reagent was purchased from Sigma-Aldrich. Matrigel was from Corning (Corning, NY, USA). Coomassie blue was purchased from Biosesang Inc. (Seongnam, Republic of Korea). Antibodies against p38, phospho-ERK1/2, ERK1/2, phospho-JNK, JNK, Act1, and β-actin were obtained from Santa Cruz Biotechnology (Dallas, TX, USA), while phospho-p38 antibody was from Cell Signaling Technology (Danvers, MA, USA).

### 4.2. Target Gene Screening and Network Construction

Phlorotannin target genes were identified using the HERB (http://herb.ac.cn/, accessed on 29 June 2024) and TCM bank (https://tcmbank.cn/, accessed on 30 June 2024) databases. Ovarian cancer-related genes were retrieved from GeneCards (https://www.genecards.org/, accessed on 29 April 2024) using the keyword “ovarian cancer” and filtered to include only protein-coding genes. Overlapping targets between phlorotannins and OVCA were selected for further analysis. Network construction was performed using Cytoscape (version 3.10.2).

### 4.3. GAD Disease, KEGG Pathway, and GO Function Enrichment Analysis

Gene disease associations (GAD), Kyoto Encyclopedia of Genes and Genomes (KEGG) pathway enrichment, and Gene Ontology (GO) analyses were performed using the DAVID database (https://davidbioinformatics.nih.gov/, accessed on 9 August 2024). GAD disease enrichment was conducted with phlorotannin targets, applying a significance threshold of *p* < 0.05. The top 10 cancer types were selected from this analysis. KEGG enrichment was performed using targets related to epithelial ovarian cancer and ovarian cancer, as identified in the GAD disease analysis, along with common targets from the phlorotannin dataset. GO enrichment was subsequently carried out for genes in the IL-17 signaling pathway, which showed the highest fold enrichment after the general “pathways in cancer” category. All results were visualized using SRplot (https://www.bioinformatics.com.cn/srplot, accessed on 19 July 2025).

### 4.4. Survival Analysis

Survival data were obtained from TCGA OVCA cohorts via UCSC Xena (https://xenabrowser.net/, accessed on 11 May 2024). Among 429 patients with IL17RA expression data, those with high expression (*n* = 212) were stratified by tumor stage (I + II vs. III + IV). Kaplan–Meier plots were generated using the ggsurvplot function in survminer (RStudio version 2025.05.1).

### 4.5. Molecular Docking and Dynamics Simulation

Phlorotannin structures (Simplified Molecular Input Line Entry System; SMILE) were retrieved from PubChem (https://pubchem.ncbi.nlm.nih.gov/, accessed on 25 August 2025) and converted to 3D structures. The IL-17RA protein structure was downloaded from the RCSB Protein Data Bank, and water molecules were removed using PyMOL (version 3.0.4). Docking was performed using standard protocols.

Molecular dynamics (MD) simulations of the phlorotannin–IL-17RA complexes obtained from molecular docking were performed using GROMACS 2023.2. The AMBER99SB force field was applied to IL-17RA, and the General Amber Force Field (GAFF) to the phlorotannins. Each complex was placed in a cubic TIP3P water box with a 1.0 nm margin, and counterions were added for neutralization. Energy minimization was conducted using the steepest descent algorithm, followed by 100 ps NVT equilibration at 300 K with the V-rescale thermostat and 100 ps NPT equilibration at 1 bar with the Parrinello–Rahman barostat. Production runs were performed for 100 ns with a 2 fs integration step. Trajectory stability was assessed by root mean square deviation (RMSD) using GROMACS tools, with results averaged in 5 ns intervals and visualized in R (ggplot2, version 2025.05.1). The most stable simulation segment was defined as the top 50% of intervals with the lowest RMSD standard deviation and minimal change from preceding segments. Binding interactions during the stable period were analyzed using LigPlot+ (version 2.2) and visualized in PyMOL.

### 4.6. Cell Culture

SKOV3 and A2780 OVCA cells were maintained in RPMI 1640 medium containing 5% FBS, penicillin, and streptomycin in a humidified 5% CO_2_ atmosphere at 37 °C. THP-1 cells were cultured in RPMI 1640 medium with 10% FBS, antibiotics, and 0.05 mM 2-mercaptoethanol. Differentiation into macrophages was induced by 100 nM PMA for 24 h, followed by polarization into TAM-like cells using conditioned medium from SKOV3 cells for 48 h.

### 4.7. MTT Assay

Cell viability was performed using a 3-(4,5-dimethylthiazol-2-yl)-2,5-diphenyltetrazolium bromide (MTT) assay. SKOV3 (0.7 × 10^5^ cells/well) and A2780 (1.3 × 10^5^ cells/well) cells were seeded in 96-well plates. After 24 h, the cells were treated with various concentrations of phlorotannins for 48 h, followed by incubation with 5 mg/mL MTT for 4 h. The medium was then removed, and the resulting formazan crystals were dissolved in 50 μL dimethyl sulfoxide (DMSO). Absorbance was measured at 560 nm.

### 4.8. Wound-Healing Assay

The wound-healing assay was conducted to evaluate SKOV3 cell migration using a previously established protocol, with minor modifications to accommodate the experimental conditions of this study [40]. The cells were seeded in 24-well plates and cultured in RPMI-1640 medium for 24 h to form a confluent monolayer. A straight scratch was made in the cell monolayer using a 10 μL pipette tip, generating a cell-free gap. Detached cells and debris were removed by washing with PBS, after the cells were incubated in FBS-free RPMI-1640 medium containing dieckol (10, 20, or 40 μM), 8,8′-bieckol, or 7-phloroeckol (20, 40, or 80 μM), or PBS vehicle control. Cell migration into the wound area was observed at 24 and 48 h using a microscope (Carl Zeiss, Oberkochen, Germany), and migration distances were quantified using ImageJ software (National Institutes of Health, Bethesda, MD, USA; version 1.53).

### 4.9. Transwell Invasion Assay

The transwell invasion assay was conducted using a previously established protocol as described in our prior study [41]. SKOV3 cells (1.1 × 10^5^ cells/well) were seeded into 6-well plates and treated with dieckol (10, 20, or 40 μM), 8,8′-bieckol, or 7-phloroeckol (20, 40, or 80 μM). In inhibitor experiments, ERK inhibitor PD98059 (10 μM) was applied 4 h before phlorotannin treatment [42]. The cells were then transferred to a transwell insert (8 μm pore size, PVPF filters) pre-coated with Matrigel (Corning) in RPMI-1640 medium supplemented with 1% FBS. The lower chamber contained RPMI-1640 medium supplemented with 10% FBS as a chemoattractant. After 48 h of incubation, the cells that had invaded the lower membrane surface were fixed with methanol for 10 min and stained with 0.2% crystal violet for 20 min. Non-invading cells on the upper membrane surface were removed with a cotton swab. Invaded cells were counted in five randomly selected microscopic fields per filter under an inverted microscope and quantified using ImageJ software.

### 4.10. Gelatin Zymography

Conditioned media from SKOV3 cells treated with phlorotannins for 48 h in serum-free medium were concentrated using a Microcon Centrifugal Filter (Merck Millipore, Darmstadt, Germany) and separated on 8% SDS-PAGE gels containing 1% gelatin. The gels were washed twice for 30 min with 2.5% Triton X-100 (Sigma-Aldrich) to remove SDS and incubated in collagenase buffer at 37 °C for 24 h. Gels were stained with Coomassie blue for 1 h and destained for 20 min to visualize gelatinolytic activity.

### 4.11. Western Blot Analysis

Cells were lysed in buffer (Intron Biotechnology, Seoul, Republic of Korea) supplemented with protease inhibitor cocktail, PMSF, and phosphatase inhibitor cocktail. Protein samples were mixed with SDS sample buffer, boiled at 95 °C for 5 min, separated on 8% or 12% SDS-PAGE gels, and transferred to nitrocellulose membranes. Membranes were blocked with 5% skim milk for 30 min, washed in TBS containing 0.1% Tween-20, and incubated overnight at 4 °C with primary antibodies diluted 1:1000 in 5% skim milk. After three washes in TBS-T, membranes were incubated with secondary antibodies (1:1000) for 2 h at room temperature, washed again, and developed using enhanced chemiluminescence (ECL) solution (Abclon, Seoul, Republic of Korea). Signals were detected with a LuminoGraphIII Lite imaging system (Atto, Tokyo, Japan).

### 4.12. Real-Time RT-PCR

Total RNA was extracted using Trizol Reagent (Invitrogen, Carlsbad, CA, USA) according to the manufacturer’s instructions. First-strand cDNA was synthesized from 500 ng total RNA using a First-Strand cDNA Synthesis Kit (Enzynomics, Daejeon, Republic of Korea). Real-time RT-PCR was performed with TB Green Premix Ex Taq™ (TaKaRa, Kyoto, Japan) under the following cycling conditions: 95 °C for 10 s, 58 °C or 60 °C for 10 s, and 72 °C for 10 s, for a total of 45 cycles. Primer sequences are listed in Table S7. The cycle threshold (Ct) values of target genes were normalized to the Ct value of the housekeeping gene APP, which was selected based on its stable expression in ovarian cancer cells and macrophages under the experimental conditions used in this study, consistent with recommendations for model-specific validation of reference genes [43].

### 4.13. Enzyme-Linked Immunosorbent Assay (ELISA)

The concentrations of IL-10, VEGF, and IL-17A in TAMs-conditioned medium were measured using commercial ELISA kits (Koma Biotech, Inc., Seoul, Republic of Korea) following the manufacturer’s protocols.

### 4.14. Statistical Analysis

Data are presented as mean ± SD from at least three independent experiments. Statistical analyses were performed using one-way analysis of variance (ANOVA) followed by Tukey’s multiple comparison test to determine differences between groups. A *p*-value < 0.05 was considered statistically significant. In all figures, “*” indicates *p* < 0.05 compared to the control group, while “#” indicates *p* < 0.05 compared to the specified comparison group. All statistical analyses were conducted using GraphPad Prism software (GraphPad Software, San Diego, CA, USA; version 10.2.3).

## 5. Conclusions

This study demonstrates that phlorotannins from *E. cava* act as bioactive marine polyphenols that modulate IL-17RA–Act1 and MAPK signaling, suppress ovarian cancer invasion, and reshape the tumor microenvironment. Structural diversity among dieckol, 7-phloroeckol, and 8,8′-bieckol confers differential engagement with receptor complexes and downstream pathways, highlighting the importance of chemical architecture in determining biological outcomes. By simultaneously targeting multiple signaling nodes, phlorotannins exemplify the potential of marine bioactive compounds to act as natural regulators of signaling network homeostasis, supporting further exploration for therapeutic applications in inflammation-associated malignancies.

## Figures and Tables

**Table 1:** Cytotoxicity data (IC_50_, μM) of three phlorotannins.

Phlorotannins	IC_50_ ^1^ (μM)	
	SKOV3	A2780
Dieckol	88.3 ± 4.6	79.3 ± 0.4
8,8′-Bieckol	95.0 ± 4.2	88.1 ± 0.7
7-Phloroeckol	137.3 ± 6.1	135.2 ± 1.3

^1^ Tables IC_50_ values were determined after 48 h treatment of OVCA cells with phlorotannins (dieckol, 8,8′-bieckol, or 7-phloroeckol). Values are presented as mean ± SD (*n* = 3).

**Table 2:** Molecular docking results of IL-17RA with phlorotannins.

	Box_Center (x, y, z)	Affinity (kcal/mol)	Ligand Efficiency (kcal/mol)	Residue Information
Dieckol	2.14, 0.92, −12.37	−8.8	−0.16	Asp103, Ala104, Ser105, His163
8,8′-Bieckol	2.05, −3.32, −10.91	−8.5	−0.16	Asp103, Gln101, Leu107, Ser105, Thr102
7-Phloroeckol	2.55, 1.29, −11.63	−7.8	−0.22	Asp103, His163, Thr102

## Data Availability

The original contributions presented in this study are included in the article/Supplementary Material. Further inquiries can be directed to the corresponding author.

## Supplementary Materials

The following supporting information can be downloaded at: https://www.mdpi.com/article/10.3390/md24010012/s1, Figure S1: Stable binding modes of phlorotannins with IL-17RA during molecular dynamics simulations; Figure S2: Effects of p38 and JNK inhibition on the anti-invasive activity of phlorotannins in SKOV3 cells; Figure S3: Role of ERK1/2 signaling in phlorotannin-induced TIMP1 expression in SKOV3 cells; Figure S4: Suppression of RANTES and MCP-1 expression by phlorotannins in SKOV3 cells; Table S1: Target genes of phlorotannins identified by GAD-disease analysis; Table S2: Top cancer types associated with phlorotannin targets based on cancer-only GAD-disease analysis; Table S3: Overlapping genes between phlorotannin and OVCA targets identified via HERB and GeneCards databases; Table S4: KEGG pathway enrichment of 18 genes overlapping between phlorotannins and OVCA; Table S5: GO analysis of “IL-17 signaling pathway”-associated genes; Table S6: Functional roles of 18 genes in ovarian cancer and their association with IL-17 signaling [44,45,46,47,48,49,50,51,52,53,54,55,56,57,58,59,60,61,62,63,64,65,66,67,68,69,70,71,72,73,74,75,76,77,78,79,80,81,82,83,84]; Table S7: Primer sequences used for quantitative real-time RT-PCR.

## Abbreviations

AKT	Protein kinase B
AR	Androgen receptor
BCL2	BCL2 apoptosis regulator
CCNA2	Cyclin A2
CDK2	Cyclin-dependent kinase 2
CHEK1	Checkpoint kinase 1
CTNNB1	Catenin beta 1
DMSO	Dimethyl sulfoxide
DPP4	Dipeptidyl peptidase 4
*E. cava*	*Ecklonia cava*
ERK1/2	Extracellular signal-regulated kinase 1 and 2
ESR1	Estrogen receptor 1
ESR2	Estrogen receptor 2
FBS	Fetal bovine serum
GAD	Genetic association database
GAFF	General amber force field
GO	Gene ontology
HMOX1	Heme oxygenase 1
IL-10	Interleukin-10
IL-17	Interleukin-17
IL-17RA	Interleukin-17 receptor A
IL-6	Interleukin-6
JNK	c-Jun N-terminal kinase
KEGG	Kyoto Encyclopedia of Genes And Genomes
MAPK	Mitogen-activated protein kinase
MAPK1	Mitogen-activated protein kinase 1
MCP-1	Monocyte chemoattractant protein-1
MD	Molecular dynamics
MMPs	Matrix metalloproteinases
MMP1	Matrix metalloproteinases 1
MMP2	Matrix metalloproteinases 2
MMP3	Matrix metalloproteinases 3
MMP9	Matrix metalloproteinases 9
MMP13	Matrix metalloproteinases 13
MTT	3-(4,5-dimethylthiazol-2-yl)-2,5-diphenyltetrazolium bromide
NCOA1	Nuclear receptor coactivator 1
NF-κB	Nuclear factor-kappa B
NOS2	Nitric oxide synthase 2
OVCA	Ovarian cancer
p38	p38 mitogen-activated protein kinases
PI3K	Phosphatidylinositol 3-kinase
PMA	Phorbol 12-myristate 13-acetate
PMSF	Phenylmethylsulfonyl fluoride
PPARG	Peroxisome proliferator-activated receptor gamma
PTGS2	Prostaglandin-endoperoxide synthase 2
RANTES	Regulated on activation, normal T cell expressed and secreted
RMSD	Root mean square deviation
ROS	Reactive oxygen species
TAMs	Tumor-associated macrophages
TCGA	The Cancer Genome Atlas
TIMPs	Tissue inhibitors of metalloproteinases
TME	Tumor microenvironment
TRAF6	Tumor necrosis factor receptor-associated factor 6
VEGF	Vascular endothelial growth factor
