# Supplementary material for: Phlorotannins from Ecklonia cava Regulate Dual Signaling Pathways, IL-17RA/Act1 and ERK1/2, to Suppress Ovarian Cancer Progression and Tumor-Associated Macrophage Activation
Source: Mar Drugs. 2025 Dec 24;24(1):12. doi: 10.3390/md24010012 (PMC12843022; doi:10.3390/md24010012)
Supplement: Supplementary file 1 [file marinedrugs-24-00012-s001.zip › Supplementary Figures.pdf]

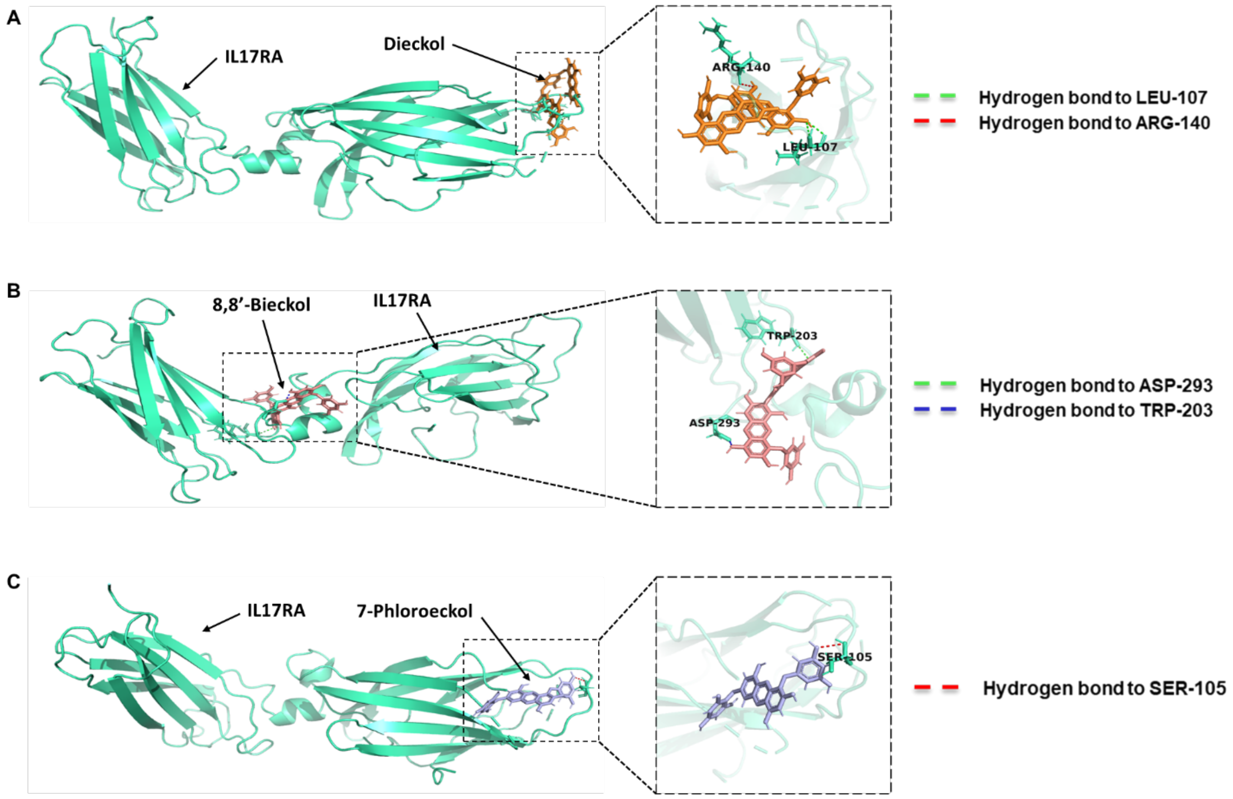

Figure S1. Stable binding modes of phlorotannins with IL-17RA during molecular dynamics simulations

(A–C) Representative binding conformations of dieckol, 8,8′-bieckol, and 7-phloroeckol with IL-17RA during the most stable phase of molecular dynamics simulations. Binding stability was determined by RMSD analysis. Hydrogen bonding patterns, hydrophobic interactions, and key contact residues are highlighted.

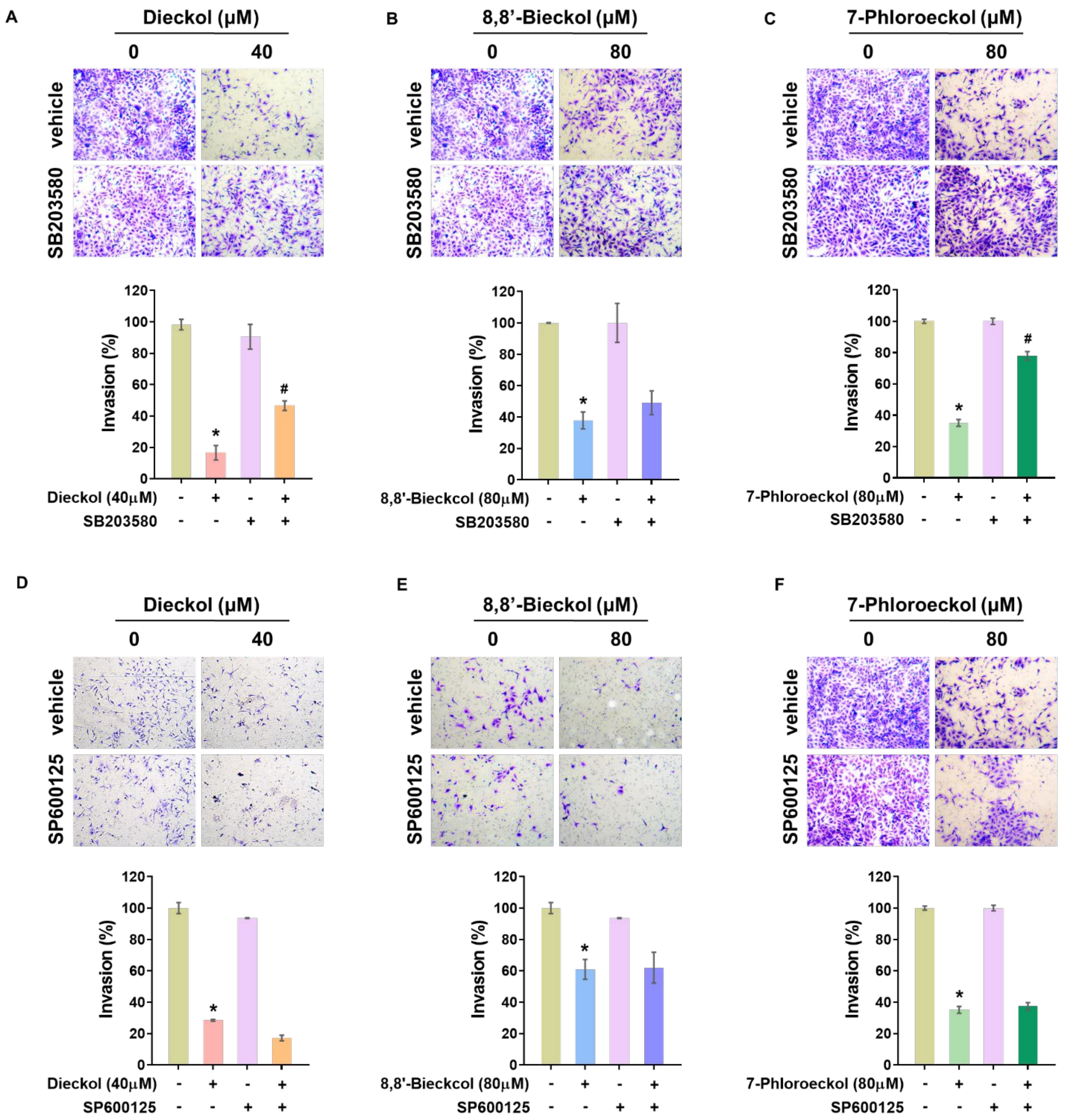

Figure S2. Effects of p38 and JNK inhibition on the anti-invasive activity of phlorotannins in SKOV3 cells

(A–C) SKOV3 cells pretreated with the p38 inhibitor, SB203580, for 4 hours were subsequently treated with dieckol (40 µM), 8,8′-bieckol (80 µM), or 7-phloroeckol (80 µM) and subjected to invasion assays. (D–F) SKOV3 cells pretreated with the JNK inhibitor, SP600125, for 4 hours

were subsequently treated with the same concentrations of phlorotannins. Invaded cells were quantified from five randomly selected microscopic fields per filter using ImageJ software. Images are representative of three independent experiments. Data are presented as the mean ± SD of three replicates. *$p < 0.05$ versus untreated group. #$p < 0.05$ compared to phlorotannin-treated group.

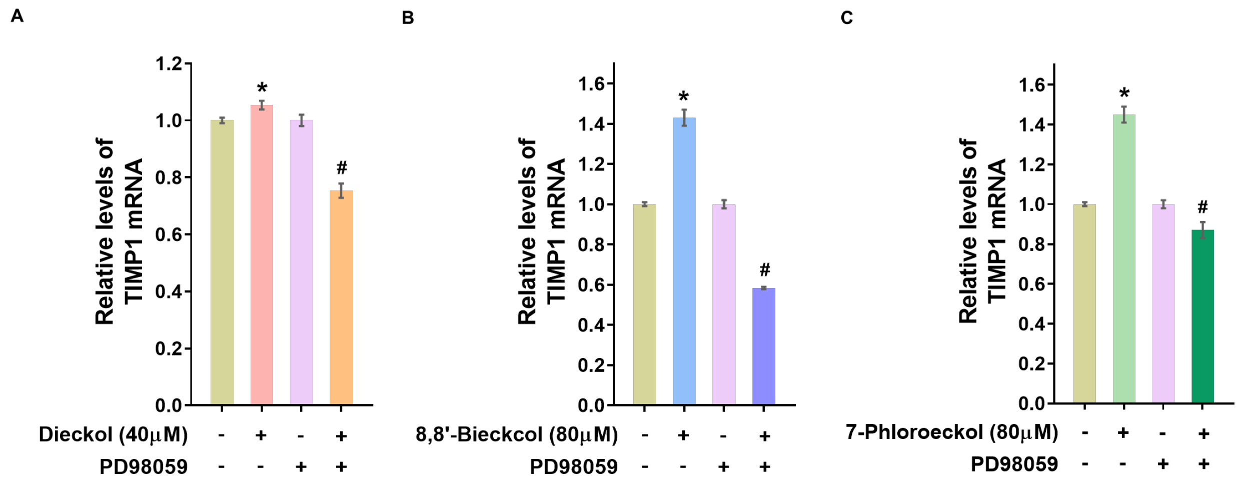

Figure S3. Role of ERK1/2 signaling in phlorotannin-induced TIMP1 expression in SKOV3 cells

SKOV3 cells were pretreated with the ERK inhibitor, PD98059, for 4 hours, followed by treatment with dieckol (40 μM), 8,8′-bieckol (80 μM), or 7-phloroeckol (80 μM). TIMP1 mRNA expression was measured by real-time RT-PCR. Data are presented as the mean ± SD of three replicates. *p < 0.05 versus untreated group. #p < 0.05 compared to phlorotannin-treated group.

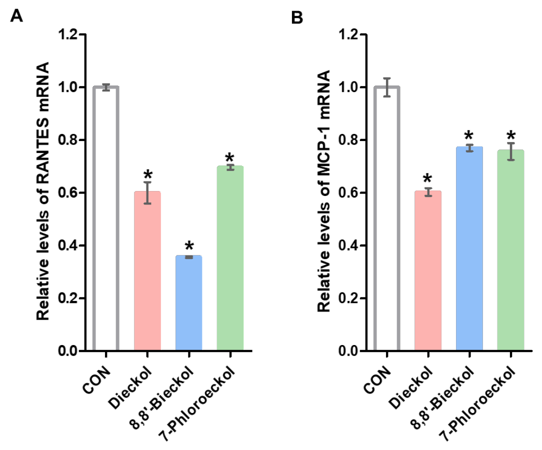

Figure S4. Suppression of RANTES and MCP-1 expression by phlorotannins in SKOV3 cells

SKOV3 cells were treated with dieckol (40 μM), 8,8′-bieckol (80 μM), or 7-phloroeckol (80 μM) for 48 hours. mRNA expression of RANTES and MCP-1 was analyzed by real-time RT-PCR. Data are presented as the mean ± SD of three replicates. *$p < 0.05$ versus untreated group.
